# Nutrition interventions in the first 1000 days and long-term health outcomes: a systematic review

**DOI:** 10.1038/s41390-025-04215-6

**Published:** 2025-07-18

**Authors:** Anna Xu, Kathrin Guerlich, Berthold Koletzko, Veit Grote

**Affiliations:** 1 Division of Metabolic and Nutritional Medicine, Department of Pediatrics, Dr. von Hauner Children’s Hospital, LMU University Hospital, LMU University Hospital, Munich, Germany; 2 German Center for Child and Adolescent Health, site, Munich, Germany; 3 University of Oxford, Nuffield Department of Primary Care Health Sciences and Department of Continuing Education, Oxford, UK

## Abstract

**Background:**

Nutrition in early life can influence health in later life. This systematic review evaluated the impact of nutrition intervention programs in the first 1000 days of life on long-term cardio-metabolic, respiratory, and mental health and dietary behaviour in children.

**Methods:**

Randomized controlled trials (RCTs) from high-income countries were searched in four databases and two trial registries in March 2020, and updated in November 2022. Risk of bias was evaluated using the Cochrane Risk of Bias tool and certainty of results using the GRADE considerations. Results were synthesized narratively.

**Results:**

Sixty-three articles covering 20 interventions were included. Most interventions began in early infancy (<6 months of age), lasted 12–36 months and had follow-ups under five years. Initial results showed some positive impact of interventions on cardio-metabolic health (three RCTs). No effect was found on mental health (four RCTs), while no RCT reported on respiratory health. Interventions showed some improvements on dietary behaviour, with mixed results across studies.

**Conclusion:**

Trials on early nutritional interventions seldom report health outcomes with long-term follow-ups. There is some evidence of a positive impact on cardio-metabolic health and dietary behaviour.

**Impact:**

- In the assessed studies, nutritional interventions in the first 1000 days of life mainly had short follow-ups and reported heterogeneous outcomes. There was some evidence that nutritional interventions can improve children’s dietary behaviour and cardio-metabolic health, while there was no effect on mental health.
- We provide a systematic review on the impact of early nutrition intervention programs on long-term health outcomes in children showing research gaps for intervention studies with long-term and clinically relevant outcomes.
- Understanding the effectiveness and implementation of early nutritional interventions provides insights for preventative population health and affects future intervention planning.

## Introduction

Early life is an important window of opportunity to improve health.^1^ There is growing research on the associations between exposures in the prenatal and early postnatal period with physical, cognitive and emotional developments, and the risk of disease later in life.^1,2^ Observational studies have demonstrated associations between early birth outcomes such as preterm birth or high and low birth weights with increased risk of obesity, cardiovascular disease, asthma, and mental health disorders through life.^2^ Despite supportive evidence from observational studies there is ongoing challenge from translating these findings into impactful lifestyle interventions.

Narrative and scoping reviews revealed that the most studied lifestyle exposures include nutrition, physical activity, smoking, and alcohol consumption.^2^ Nutrition and diet are deeply investigated topics with known pivotal impacts on overall health and development.^3,4^ There is strong observational evidence on the association of poor nutrition and increase of obesity, cardiovascular diseases, diabetes, and some cancers.^5^ Diet and nutrition are modifiable factors targeted by numerous public health projects.^5,6^ However, existing studies focus predominantly on low-income and high-risk populations, while the focus in high-resource settings is concentrated on obesity outcomes.^7,8^ There is also a lack of results on the clinical long-term impact of these programs.^7,8^

From the social and developmental perspective, there is a generational effect of health improvement by targeting parents.^9,10^ It is hypothesized that in the prenatal and preconception periods parents are more sensitive to lifestyle improvements due to greater incentives and increased awareness of the importance of early exposure on later health of the offspring.^9,10^ There is unclear evidence on the impact of using parents as agents of change in improving the effectiveness of dietary interventions, but establishing healthy behaviors early in life and promoting change in parents is nonetheless a potential additional benefit of implementing interventions in the critical period.^11^

Therefore, this systematic review aims to examine the existing evidence surrounding the impact of nutrition intervention programs in the first 1000 days of life on long-term child cardio-metabolic (aspects of cardiovascular and metabolic health affecting heart and metabolic function, e.g. blood pressure, cholesterol levels, glucose regulation), respiratory and mental health and dietary behavior, and gain understanding of their efficacy and stage of development. The four key questions are: (1) What is the evidence on the long-term health impacts of early nutrition interventions? (2) What type of interventions are used to promote recommended nutrition and diet behavior? (3) What is the quality and design of these trials? (4) What should further research focus on and what recommendations can be made?

As this review was part of the European Horizon 2020 LifeCycle project (2017-2022), the main outcomes and exposures as defined in the project were the basis to our objectives, defined in the PICOT.^12^

## Methods

This systematic review was conducted according to the PRISMA guidelines (Preferred Reporting Items for Systematic reviews and Meta-Analyses).^13^ The review protocol has been registered in PROSPERO (ID: CRD42020167893).

### Eligibility criteria

Table 1 summarizes the PICOT framework for the inclusion criteria for this review. Randomized controlled trials (RCTs) and cluster randomized trials were included. This review focused on high-income countries with health contexts comparable to that of Western Europe. Trials with healthy adults in the preconception and prenatal period as well as parents of children up to two years of age or children up to two years of age were eligible. Samples should have been reflective of their communities.

**Table 1:** Summary of PICOT for eligibility criteria.

Study Design: Randomized controlled trials (RCTs), Cluster randomised trials	
**Population**	Healthy adults: preconception, prenatal Children up to 2 years of age Parents of children up to 2 years of age
**Intervention**	Nutrition programs targeted at diet lifestyle and improving nutritional knowledge and healthy dietary behaviors
**Comparator**	Standard Care, alternative non-nutrition program
**Outcomes**	Clinical outcomes in the child: cardio-metabolic, respiratory, mental health and development Behavioral outcomes in the child: diet practices, nutritional intake, health knowledge
**Time**	Minimum 12 months follow-up

Nutrition interventions were defined as programs focused on improving diet behavior, nutritional intake, providing support, recommendations, or education to promote healthy dietary choices, lifestyles, and expand nutrition knowledge. Interventions could be a composite of different lifestyle factors (e.g. counseling on physical activity, screen time or sleep) with a nutrition component (e.g. counseling on healthy nutrition, meal planning) or target specific diet choices (e.g. reduce sodium intake). Supplements, diet manipulations, and breastfeeding interventions alone were excluded. The control groups should have received standard community care or a non-nutrition comparator program (e.g. physical activity, composite lifestyle intervention not including a nutrition component). No restrictions were applied to intensity or duration of intervention.

Clinical long-term outcomes in the offspring were the focus of this review (Supplement I Table S1). Primary outcomes were endpoints or clinical measurements that have significant associations with cardio-metabolic health (cardiovascular diseases (e.g. hypertension, atherosclerosis), cardiovascular measures (e.g. blood pressure, pulse rate), metabolic diseases (e.g. diabetes)), respiratory health (respiratory diseases (wheezing, asthma, COPD)), and mental health (mental health diagnoses (e.g. Attention Deficit Hyperactivity Disorder, Autism-Spectrum Disorder, internalizing and externalizing behavior)). Influence on child diet behavior (dietary intake (e.g. portions of fruits, sodium intake), diet quality (e.g. variation of intake), nutrition knowledge) were also included. Studies that focused solely on obesity, overweight and adiposity as outcomes were excluded. Studies needed to follow-up for 12 months or longer to ensure focus on long-term outcomes.

### Search strategy

The search strategy was developed for MEDLINE (PubMed) and then subsequently translated for EMBASE, CINAHL, and the Cochrane Central Register for Controlled Trials (CENTRAL) (Supplement II). Cochrane low-middle income countries filters were applied, which were based on the World Bank list of countries, to exclude low-middle income countries from our search results.^14^ To ensure literature saturation, reference lists of included studies were hand searched. Authors’ personal repositories were also searched to capture relevant material.

Recent and ongoing trials were searched for in the International Standard Randomised Controlled Trial Number (ISRCTN) Registry and on ClinicalTrials.gov. As studies were identified, additional cited and citing articles were examined.

The search was conducted in March 2020, and further updated in November 2022. No language or date limitations were applied.

### Study selection

Identified records were uploaded into reference manager EndNote (Version X8 and 20) and duplicates were removed. Titles, abstracts, and full texts were independently screened by two reviewers (AX, MV, KG, LR).

### Data extraction

Data extraction was completed following the Cochrane data extraction template with Covidence^15^ by two independent reviewers (AX, KG or LR). Data items extracted included authors, year of publication, country, trial design, study aim, enrollment design, sample size, participant characteristics (age, sex, ethnicity), intervention characteristics (type, duration, target group, design, comparator), duration of follow-up, assessed outcomes and collection methods, and funding.

Any discrepancies in study selection or data extraction were resolved through discussion or consultation of a third independent reviewer (VG).

### Risk of bias assessment

Risk of bias assessment was conducted independently by two reviewers (AX, KG or LR) using the Cochrane risk of bias tool.^16^ For each domain in the tool, information from the studies was presented, including verbatim quotes, and assigned a ‘high’ or ‘low’ risk rating. If there was insufficient data, the risk of bias was assigned as ‘unclear’. Risk of bias was assessed using the outcomes relevant to this review rather than the original study, and at the latest follow-up reported. Discrepancies were resolved through discussions.

### Certainty of results assessment

We used the five GRADE considerations (study limitations, consistency of effect, imprecision, indirectness, and publication bias) to assess the certainty of the evidence related to the studies that contributed data to the narrative synthesis for the groups of prespecified outcomes.^17^ We assessed the certainty of evidence as high, moderate, low, or very low. We used the methods and recommendations described in Murad et al. for rating the certainty in evidence in the absence of meta-analyses.^18^

We justified all decisions for the certainty assessment using footnotes, and a detailed analyses by outcome groups is available in Supplement III.

### Synthesis of results

Extracted data was synthesized narratively. A meta-analysis was not conducted due to methodical and statistical heterogeneity of data and incomplete numerical reporting of outcomes. Outcomes from multiple reports of the same study were selected according to the latest follow-up measurements.

## Results

### Identification of studies

**Fig. 1:**
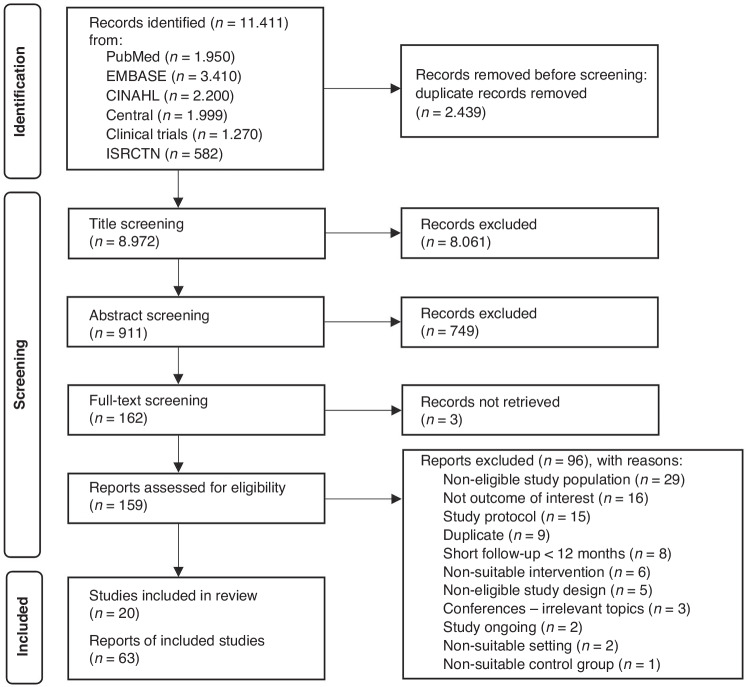
PRISMA flow diagram of the literature search and selection process. *From:* Page MJ, McKenzie JE, Bossuyt PM, Boutron I, Hoffmann TC, Mulrow CD, et al. The PRISMA 2020 statement: an updated guideline for reporting systematic reviews. BMJ 2021;372:n71. 10.1136/bmj.n71.

Of 11,411 records identified, 162 articles remained for full-text screening. Full texts for three reports were unable to be retrieved after attempts to contact the study authors^19–21^ resulting in 159 articles. Overall, 63 reports met inclusion criteria (Fig. 1).^22–84^ The full list of exclusion reasons is listed in Fig. 1 and in Supplement I Table S2.

### Study characteristics

The 63 included reports corresponded to 20 distinct interventions, including 38 reports from one longitudinal RCT spanning 26 years.^33–70^ Details on characteristics of the included studies are presented in Table 2 and in Supplement IV.

**Table 2:** Characteristics and results of included studies

Study ID/Report ID	Country	Intervention target / duration	Aim	Age at start	N at baseline	Outcomes reported	Cardio-metabolic health	Mental health	Diet Behavior
**Multiple Nutrition Intervention**									
**Beinert et al**.^22^ **(First food for infants)** Overby et al.^72^	Norway	Infancy 2 days	Improve child eating behavior/ parental feeding practices	4–6 mos.	110	Food Intake, Food Preference, Supplementation Lipid Profile	^a^24 mos.		^a^24 mos.
**Daniels et al**.^26^ **(BLISS study)**	New Zealand	Pregnancy 10 mos.	Obesity prevention	Third trimester of pregnancy	206	Nutrient Intake			^b^12 mos.
**De Franchis et al**.^82^	Italy	Infancy 12 mos.	Improve adherence to Mediterranean diet	4–6 mos.	394	Dietary Patterns			^a^18 mos.
**Helle et al**.^29^ **(Early Food for Future Health)**	Norway	Infancy 6 mos.	Improve child eating behavior/ parental feeding practices	3–5 mos.	718	Food Intake, Eating Behaviors			^c,g^24 mos.
**Hoppu et al**.^31^	Finland	Pregnancy 21 mos.	Impact on cardiovascular health	First trimester of pregnancy	256	Nutrient Intake			^d,h^12 mos. to 4 y
**Roed et al**.^78^	Norway	Infancy 6 mos.	Improve child eating behavior	11 mos.	298	Food Intake			^b^2 y
**Lifestyle Intervention**									
**Campbell et al**.^23^ **(InFANT Study)**^f^ Hesketh et al.^30^, Spence et al.^74^	Australia	Infancy 15 mos.	Obesity prevention	4 mos.	542	Dietary Patterns Food Intake			^e^18 mos. ^e^5 y
**Döring et al**.^27^ **(PRIMROSE)**^f^	Sweden	Infancy 39 mos.	Obesity prevention	5–6 mos.	1369	Food Intake			^a,^^e^4 y
**Fangupo et al**.^28^ **(POI.nz)** Taylor et al.^73^	New Zealand	Infancy 18 mos.	Improve child eating behavior/ parental feeding practices	4–6 weeks	802	Food Intake, Nutrient Intake Dietary Patterns Self-regulation abilities		^b^3.5 y	^b^2 y ^b^3.5 y
**Niinikoski et al**.^33^ **(STRIP Study)** Rask-Nissilä et al.^56^ Talvia et al.^65^ Räsänen et al.^55^ Niinikoski et al.^46^ Ruottinen et al.^60^ Niinikoski et al.^47^ Oranta et al.^67^ Niinikoski et al.^48^ Kaseva et al.^41^ Lapinleimu et al.^42^ Nupponen et al.^49^ Lehtovirta et al.^43^ Nuotio et al.^50^ Matthews et al.^44^ Laitinen et al.^68^ Rovio et al.^70^ Pahkala et al.^69^	Finland	Infancy 26 years	Atherosclerosis and coronary heart disease prevention	7 mos.	1062	Nutrient Intake, Food Intake, Dietary Patterns, Food Knowledge Blood pressure categories Metabolic syndrome Continuous blood pressure, Systolic blood pressure, Diastolic blood pressure Cardiovascular measures Lipid Profile Biochemical Profile Cognitive Development Psychological wellbeing, School performance	^e^15 to 20 y ^e^7 mos. to 15 y ^b^26 y ^b^11 y, 11 to 19 y ^a,e^15 to 19 y ^a^26 y ^a,e^9 to 19 y ^e^26 y ^e^15 to 20 y	^b^5, 26 y ^b^14, 20 y	^b^9, 26 y ^a,e^9, 10, 19 y ^d,i^20 y ^a^20 y
**O’Sullivan et al**.^71^	Ireland	Pregnancy 6 years	Improve cognitive Development	Pregnancy	233	Food Intake Cognitive Development		^b^3 y	^b^3 y
**Ruggiero et al**.^83^	United States	Infancy 2.5 years	Improve child dietary behaviour	10–14 days	279	Eating Behaviours			^e^2.5 y
**Van Vliet et al**.^84^	Netherlands	Infancy 12 mos.	Improve child dietary behaviour	4–6 mos.	246	Food Intake			^b^2 y
**Verbestel et al**.^75^^,*f*^	Belgium	Infancy 1 year	Obesity prevention	9–24 mos.	203	Food Intake			^b^9 to 24 mos.
**Wen et al**.^77^	Australia	Pregnancy 26 mos.	Obesity prevention	Third trimester of pregnancy	667	Food Intake Quality of Life, Physical/Emotional/Social/School Functioning		^b^5 y	^b^5 y
**Wen et al**.^79^	Australia	Pregnancy, infancy 2 years	Improve child eating behaviour/ parental feeding practices	Third trimester of pregnancy	1155	Food Intake			^b^2 y
**Nutrition Education**									
**Childs et al**.^24^	United Kingdom	Infancy 18 mos.	Anaemia prevention	Birth	1000	Dietary Patterns			^b^18 mos.
**Daniels et al**.^25^ **(NOURISH RCT)** Magarey et al.^32^	Australia	Infancy 18 mos.	Improve child eating behaviour/ parental feeding practices	2–7 mos.	698	Food Intake, Eating Behaviour, Food Preferences, Dietary Patterns			^e^24 mos. ^b^24 mos. to 5 y
**Watt et al**.^76^	United Kingdom	Infancy 12 mos.	Improve child eating behaviour/ parental feeding practices	10 weeks	312	Food Intake, Nutrient Intake			^d,j^18 mos.
**Wozniak et al**.^80^ Wozniak et al.^81^	Poland	Infancy 12 mos.	Impact on child iron and metabolic status	Under 8 weeks	203	Nutrient Intake Lipid Profile	^e^1 y		^a,e^1 y

*mos* months, *y* year

^a^Increased favorable behavior

^b^No difference

^c^Favored control

^d^Conflicting significant results (had both favored control and intervention)

^e^Decreased unfavorable behavior

^f^Cluster RCT

^g^Favored control group for emotional overeating score and food responsiveness score.

^h^Favored control group for mean vitamin C intake (95% CI), I: 66 mg/day (62–69), C: 75 mg/day (68–81), treatment effect (9.2 (1.6 to 16.9), p = 0.017, however both groups had over recommended daily intake for vitamin C.

^i^Favored control group for decreasing sodium intake, ß-coeff (95%-CI): 55.00, (24.40 to 85.60 mg/day), p < 0.001.

^j^Favored control for decreased consumption of chips and other potatoes.

Intervention Types: 1) nutrition education 2) meal planning (theoretical/practical), 3) complementary food, 4) supplements, 5) multiple - combination of 1–4, 6) lifestyle interventions - combination of 1–4 with other lifestyle related interventions (e.g., promoting physical activity, parenting education, healthy behaviors). All controls used were standard care unless indicated.

### Design

Of the 20 interventions, nearly all were RCTs (*n* = 17); three studies were cluster RCTs.^23,27,75^

### Population

The included studies were from 12 countries: Australia (*n* = 4), Norway (*n* = 3), United Kingdom (*n* = 2), New Zealand (*n* = 2), Finland (*n* = 2), Ireland (*n* = 1), Sweden (*n* = 1), Poland (*n* = 1), United States (*n* = 1), Netherlands (*n* = 1), Italy (*n* = 1), and Belgium (*n* = 1). Study participants included parents during pregnancy (*n* = 5)^26,31,71,77,79^ or parents with their infants (*n* = 15).^22–25,27–29,33,75,76,78,80,82–84^ The total number of participants varied from 110 to 1369.

All studies had a majority of European or Caucasian participants except for one study, which included mostly Asian participants.^24^ Four studies^24,71,76,77^ contained a large proportion of low socioeconomic status participants. In the 16 remaining studies, parents/mothers were largely well-educated, married, and non-smokers. In the studies, which recruited mothers, it was unknown whether the intervention was delivered to only mothers or both parents.

### Intervention characteristics

#### Start, duration, and follow-up

Nearly all interventions focused on the infancy period, defined as the timeframe from birth to 24 months of age (*n* = 15).^22–25,27–29,33,75,76,78,80,82–84^ Most studies (*n* = 12) started intervention before 6 months of age^22–25,27–29,76,80,82–84^ and three studies started intervention between 6-12 months of age.^33,75,78^ Five interventions began in pregnancy, of which, three studies started in the third trimester,^26,77,79^ one study started in the first trimester,^31^ and one study did not clarify the exact start of the intervention.^71^ No study targeted the preconception period.

The majority of interventions (*n* = 14) lasted between 12 and 36 months.^23–25,27,28,31,75–77,79,80,82–84^ The duration of three interventions was between 6 and 12 months^26,29,78^ and two interventions were longer than 5 years.^33,71^ Only one intervention implemented a one-time session which took place over two days.^22^ The longest intervention lasted 20 years, being delivered first to the parents and from seven years of age onwards directly to the children.^33^

In terms of follow-up, five studies reported outcomes at less than two years of age,^24,26,76,80,82^ 14 studies reported outcomes between 2 and 5 years of age,^22,23,25,27–29,31,71,75,77–79,83,84^ and one study reported outcomes at 26 years of age.^33^ However, not all outcome domains were followed up to the latest age.

#### Setting, support type and delivery agent

Most of the interventions were delivered in person (*n* = 15); three in only group settings,^22,23,25^ nine with 1-on-1 sessions^24,26,31,33,71,77,82–84^ and three through combined group and individual in-person sessions.^27,28,76^ Six of the in-person studies included home visits^28,71,76,77,83,84^ and two implemented a feeding protocol for parents.^82,84^ The remaining five studies delivered the intervention through solely independent learning material in the forms of written posters (*n* = 1)^75^ or e-health interventions with online content (*n* = 2)^29,78^ or remote support such as telephone calls or texting (*n* = 2).^79,80^

Over half of the interventions (*n* = 12) were delivered by professional mentors such as nurses, dieticians/nutritionists, physicians, midwives, lactation consultants, or a trained researcher/teacher.^22–28,31,33,77,79,82,83^ Three interventions used lay (non-professional support), such as peers or other members of the community.^71,76,84^

#### Components and theoretical framework

Interventions were based on different theoretical frameworks. Three studies were based on Nordic dietary recommendations,^22,31,33^ one on Dutch dietary recommendations^84^ and one on Mediterranean diet.^82^ Three studies were targeted at preventing iron deficiency^24,26,80^ and one on healthy eating behaviors such as more frequent home cooking.^71^ However, most studies (*n* = 11) were based on social cognitive or parent support theories.^23,25,27–29,75–79,83^

Interventions were categorised into three types: (1) Nutrition education—counseling on healthy nutrition behaviors and recommendations (*n* = 4),^24,25,76,80^ (2) Lifestyle interventions—counseling on nutrition as well as other lifestyle behaviors such as parenting or feeding practices, sedentary behavior, sleep, or non-smoking counseling (*n* = 10),^23,27,28,33,71,75,77,79,83,84^ and (3) Multiple nutrition interventions—providing additional components to support healthy dietary behaviors beyond counseling, such as meal planning (theoretical through providing recipes, or practical through cooking classes), supplying complementary food items to the participants, or giving supplementation (*n* = 6).^22,26,29,31,78,82^ All studies used non-nutrition-based standard care (depending on the country) as a control group.

Results were mixed among the different intervention types and delivery methods.

#### Impact on outcomes

The 20 interventions reported outcomes in cardio-metabolic and mental health, and dietary behavior. No study reported respiratory health outcomes. Outcome variables were either reported continuous or categorical, and analyzed longitudinally or at a final timepoint. Details on the interventions and results can be found in Supplement IV and V.

#### Impact on cardio-metabolic outcomes

Three studies^22,33,80^ reported cardio-metabolic outcomes, categorised into five outcome types: (1) metabolic syndrome,^49^ (2) blood pressure,^46,48,49,69^ (3) lipid profile,^43,69,72,81^ (4) biochemical profile,^67^ and (5) cardiovascular measures.^42,51,68,69^ Of the three studies, two reported outcomes at <2 years of age.^22,80^

All three interventions found some favorable impact on cardio-metabolic outcomes as well as null effects in a subset of measurements. Overby et al. found slightly higher mean high-density lipoprotein cholesterol (HDL-C) levels in the intervention group at 24 months of age (*p* = 0.023) and a larger change in mean (SD) between 15 and 24 months of age, while the control group showed a decline (I: 0.29 (0.41) mmol/l vs. C: –0.08 (0.40) mmol/l, *p* = 0.002).^72^ Wozniak et al. found slightly lower median triglyceride levels (*p* < 0.001) and lower median (IQR) triglyceride to HDL-C ratios (I: 2.52 (1.68, 2.96) vs. C: 3.10 (2.88, 4.07), *p* < 0.001) in the intervention group at 12 months of age.^81^

The STRIP study found various benefits of decreasing unfavorable cardiovascular measures and increasing favorable lipid profile and cardiovascular measures until 26 years of age. Pahkala et al. found decreasing mean (SD) glucose levels (I: 5.00 (0.43) mmol/l vs. C: 5.07 (0.46) mmol/l, *p* = 0.40) and median (IQR) HOMA-IR levels (I: 1.44 (1.09, 1.91) vs. C: 1.62 (1.22, 2.09), *p* = 0.037) in the intervention group.^69^ The study also found small positive impact of the intervention for meeting ideal total cholesterol defined as <5.17 mmol/l (RR: 1.45, 95%CI: 1.05–2.01), and meeting optimal low-density lipoprotein cholesterol (LDL-C) levels defined as <3.0 mmol/l (RR: 1.30, 95%CI: 1.03, 1.66) in the intervention group.^69^ However, the same report also found null effect at 26 years of age in all other cardio-metabolic health metrics, including mean systolic and diastolic blood pressure.^69^ Data from the 20 years follow-up showed that the intervention group had a reduced risk for metabolic syndrome (defined as 80th/20th percentiles used as cut-off points in waist circumference, blood pressure, triglycerides, glucose, and HDL-C) (RR: 0.59, 95%CI: 0.40, 0.88), as well as a slightly reduced risk of blood pressure ≥80th%tile and blood pressure ≥85th%tile, but no difference for ≥90th%tile.^49,85^ Another report with data from 15 to 19 years of age favored intervention for decreasing risk of low ideal cardiovascular health score (RR: 1.35, 95%CI: 1.04, 1.77), however found null effect between groups in continuous ideal cardiovascular score, *p* = 0.12.^51^ Within the ideal cardiovascular score, the study specifically favored intervention for increasing occurrence of ideal cholesterol (defined as <4.40 mmol/l), and ideal blood pressure (defined as <90th percentile).^51^ Null effect was found for ideal glucose levels (<5.6 mmol/l).^51^ The cardiovascular health score was based on criteria from the American Heart Association (including smoking, BMI, physical activity, diet, cholesterol, blood pressure, plasma glucose), scoring both on a continuous range from 0 to 7 and as a dichotomous result (low ideal score: ≤3 ideal metrics).^51,86^

#### Impact on mental health outcomes

Mental health and development outcomes were reported in four studies^28,33,71,77^ with five outcome categories: (1) Self-regulation abilities,^73^ (2) Quality of Life,^77^ (3) cognitive development (e.g. speech and language skills, gross motor functioning and perception, and visual motor skills),^56^ (4) psychological well-being,^41^ and (5) school performance.^41^

All four studies found no difference in intervention effect.

#### Impact on dietary behavior outcomes

All 20 included studies reported dietary behavior outcomes and were grouped into six categories: (1) food intake, (2) nutrient intake, (3) eating behavior, (4) food preferences, (5) supplementation, and (6) other measurements (diet scores or patterns, food knowledge) used in the study.

Nearly half of the studies found null effect on all outcomes measured (*n *= 9).^24,26,28,71,75,77–79,84^

Seven studies found favorable impact on outcomes for food intake, nutrient intake, eating behavior, supplementation, and dietary scores.^22,23,25,27,80,82,83^ Overby et al. favored intervention in increasing proportion of children taking cod liver oil supplements (I: 19 (48.7%) vs. C: 3 (15.0%), *p* < 0.05), while finding null effect in all other food intake and supplementation measures.^22,72^ De Franchis et al. favored intervention for improving estimated marginal mean KidMed scores (Mediterranean Diet Quality Index for children and adolescents) (*p* < 0.001) and for higher OR of adhering to Mediterranean diet (OR: 2.80, 95%CI: 1.82, 4.3).^82^ Spence et al. favored intervention for increasing the Obesity Protective Dietary Index score at 18 months of age (ß: 1.33, 95%CI: 0.28. 2.39).^74^ Another report of the same study showed that the intervention had a small effect in reducing the intake of non-core drinks and sweet snacks at 5 years of age.^30^ Döring et al. favored intervention group for a higher intake of vegetables, less intake of sugary drinks, less intake of discretionary calories, and less intake of french fries.^27^ Daniels et al. favored intervention for decreasing the proportion of children who consumed fried potatoes (*p* = 0.04) and for less non-core beverages tried (*p* = 0.01) and more vegetables tried median (*p *= 0.008).^25^ Ruggiero et al. favored intervention for decreasing emotional overeating.^83^ Wozniak et al. favored intervention for increasing median intake of iron, fiber, and vitamin C at 1 year of age.^80^ Another report of the same study reported that the intervention group showed a decreased median intake of energy, fats, carbohydrates, and saccharose at 1 year of age.^81^

Four studies found conflicting results where control was favored for some categories.^29,31,33,76^ Watt et al. favored control group for decreasing RR (95%CI) of consumption of fries to once or less per week (*p* = 0.03), while favouring intervention for increasing RR in consumption of pears to >1 per week (*p* = 0.03).^76^ Helle et al. favored control for lower emotional overeating scores (*p* = 0.019) and food responsiveness scores (*p* = 0.026), and found null effect in all other food intake and eating behavior measures.^29^ Hoppu et al. favored control group for increasing vitamin C intake (*p* = 0.017), however both intervention and control groups had exceeded the daily recommended intake for vitamin C.^31^ The same study favored intervention for increasing intake of PUFA in girls (*p* = 0.042), but not in boys and null effect in all other nutrient intake measures.^31^ The STRIP study favored control group for lower sodium intake at 20 years of age (*p* < 0.001), however this effect was not seen at 26 years of age.^44,69^ While null effect was seen in all nutrient and food intake measures at 26 years of age, the study favored intervention at 20 years of age for higher mean diet score (*p* < 0.001), a higher favorable food diet score (*p* < 0.001) and unfavorable food diet score (*p* < 0.001), increasing nutrient intake (fiber, vitamin C, vitamin D, vitamin E, folate, iron, magnesium, zinc, and potassium intake), food intake (low-fat unsweetened dairy, vegetable-based oil fat, fish, vegetables, pulses, sprouts, fruits and berries, and fiber-rich grain products) and decreasing desserts intake.^44,69^

#### Risk of bias

**Fig. 2:**
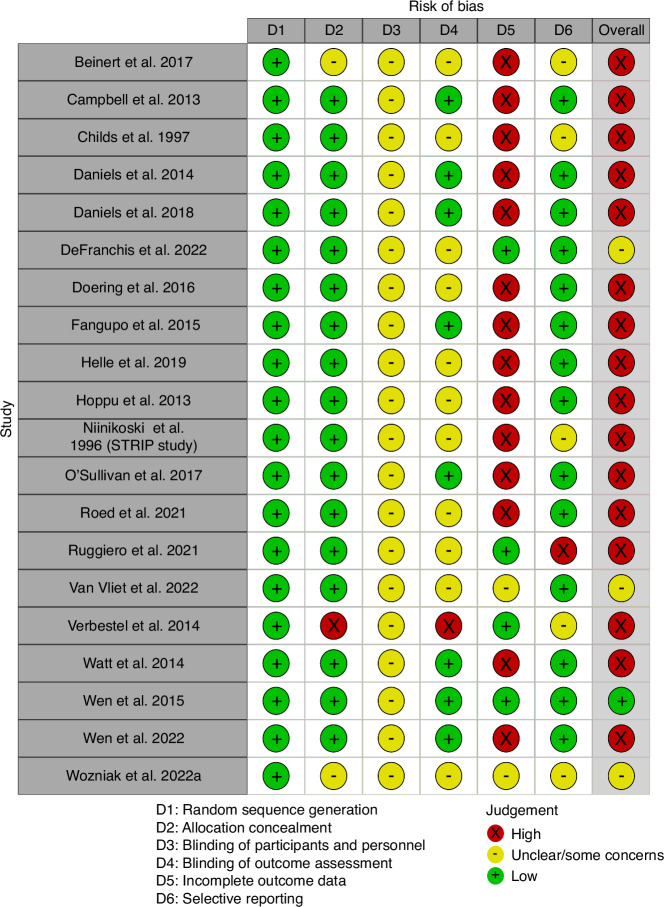
Risk of Bias table of included studies produced by the Cochrane RobVis tool.^104^

One out of the 20 studies was classified to be at low risk of overall bias (Fig. 2).^77^ Three studies were considered to have some concerns for risk of bias due to unclear reporting on blinding of outcome assessment and some concerns for attrition bias^80,82,84^ and the remaining studies were considered high risk. The blinding of participants and personnel (performance bias) was not possible due to the nature of education interventions. While this may contribute to some bias such as participants seeking other sources of nutritional education on their own, placebo effect, or personnel bias, we considered this impact to be “not high risk” as it is an inherent exposure of the education interventions. The most common source of concern was incomplete outcome data, which is a typical point of weakness for long-term trials, with 14 studies being considered “high risk” for attrition bias. We considered attrition bias to be at high risk if studies had attrition rates >30% or attrition rates >20% with unequal attrition between intervention and control groups or demographic differences in the group lost to follow-up. Finally, we considered other biases, including adherence to intervention. Most studies were scored as ‘unclear’ due to lack of reporting on adherence to intervention. An overview of risk per domain is presented in Supplement I Fig. S1.

#### Certainty of evidence

Overall, we assessed studies to provide low-certainty evidence indicating early nutrition education interventions have a small positive impact on cardio-metabolic and no effect on mental health. Compared with standard care, there is moderate-certainty evidence to indicate that these interventions have a small positive impact on dietary behavior.

The evidence for cardio-metabolic and mental health outcomes were downgraded two steps: once for borderline methodological limitations due to high risk of attrition, and a second time for serious imprecision. The evidence for dietary behavior was downgraded once for borderline imprecision and inconsistency of results (Table 3).

**Table 3:** Summary of findings for certainty of results assessment.*

Grade domain	Effect	Number of participants (studies)	Certainty of the evidence
**Cardio-metabolic Outcomes**	Most studies showed small positive impact to no effect	1375 (3 RCTs)	LOW ⊕⊕OO^a,b^
**Mental Health Outcomes**	Most studies showed no effect	2764 (4 RCTs)	LOW ⊕⊕OO^b,c^
**Dietary Behavior Outcomes**	Most studies showed small positive impact to no effect	10,755 (17 RCTs, 3 cluster RCTs)	MODERATE ⊕⊕⊕O^d^

^*^A detailed analyses by outcome groups is available in Supplement III.

^a^Serious risk of imprecision as two studies had attrition rates and borderline inconsistency with conflicting results.

^b^Borderline risk of bias due to high attrition rates.

^c^Serious imprecision was considered as there were high attrition rates, non-reported power-calculations, and ‘no effects’ reported in studies.

^d^Borderline risk of imprecision and inconsistency considered together due to high attrition rates, large number of studies with small sample sizes, small to no effects reported in studies, and some conflicting results.

## Discussion

### Summary of findings

This systematic review examined the current evidence from RCTs on nutrition interventions in early life and their impact on long-term health. In our assessed sample, interventions mostly started in infancy and lasted mainly between 12 and 36 months. Short follow-ups below 5 years of age were common. The majority of studies used in person meetings to deliver the intervention. The intensity of the interventions was heterogeneous and the content of the interventions differed, but half of them focused on combining counseling on nutrition and on a lifestyle behavior. Outcomes were highly heterogeneous and often did not examine relevant clinical endpoints. Overall, there is no strong, but some evidence that nutrition interventions in the first 1000 days of life have an impact on long-term clinical health outcomes. We can summarize that dietary behavior changes seems to be feasible and there is preliminary evidence of improvements in cardio-metabolic risk factors (based on three studies).

Overall, this systematic review showed gaps in research for long-term, clinically relevant outcomes, differing outcomes in boys and girls, identified high heterogeneity amongst early nutrition intervention studies, and insufficient evidence on efficacy of intervention in different target periods.

### Intervention characteristics

There is insufficient evidence to draw conclusions on the effectiveness of interventions targeting different periods: no study started during the preconception period and only five studies started during pregnancy.^26,31,71,77,79^

There are differences in recommendation and lifestyles in different communities, therefore future studies examining relevant clinical endpoints would increase applicability. Smaller, community-based trials can increase insight on intervention types and delivery that impact outcomes. Continued research on early life nutrition interventions and long-term health will increase prevention efficacy.

### Impact on outcomes

There were no studies reporting respiratory health outcomes. Most of the studies had five years or less follow-up time, which is too short to investigate long-term outcomes. Only one study followed up participants into adulthood. The STRIP study, was a long-term randomized controlled trial that followed participants to 26 years of age.^33^ Large scale long-term trials such as the STRIP study are optimally positioned to detect lifestyle intervention impacts on long-term health outcomes and it is crucial to be measuring clinically relevant endpoints in these longitudinal trials. However, as the STRIP study reported the most favorable results, it is also important to consider that this study had the largest sample size and used a 20-year long nutrition counseling intervention, which may have increased intervention effectiveness. Other outcomes reported were surrogate measurements for cardiovascular disease which are limited by their associations with disease occurrence in the applicability of their results to reflect the intervention effect on promoting healthy individuals.

Areas of high heterogeneity between studies were identified, especially in the reporting of mental health outcomes and dietary behaviors. Heterogeneity in the statistical analysis of outcomes occurred in studies with longer term follow ups.^31,33^ Outcomes were reported using longitudinal repeated measure analyses or solely based on endpoint measurements. Future research should consider when intervention effects over time are more beneficial than analysis of one timepoint and determine a consensus. For instance, dietary behavior outcomes over time can reflect consistency in dietary lifestyle choices and thus may be superior to dietary behavior measured at latest follow-up age only. Mean blood pressure difference between groups analysed over time may not be as relevant as blood pressure at the latest age of follow-up, unless reporting cases of hypertension.

Most of the included studies did not study relevant clinical endpoints (e.g. blood pressure, diabetes, attention deficit hyperactivity disorder), but were focused on secondary outcomes like clinical surrogate measurements (e.g. blood glucose, carotid intima media thickness, intelligence). These are generally easier to measure and collected more likely in all participants, however they do not always provide a meaningful clinical picture.

### Comparison with other studies/reviews and their findings

While there are no existing reviews that examine the long-term effects of early nutrition programs, our findings are in line with existing reviews on early nutrition interventions. In the 2018 Cochrane review of interventions for increasing fruit and vegetable consumption in children aged 5 and under, the study authors concluded that there was insufficient data available to assess long-term effectiveness of interventions.^87^ A 2017 systematic review and meta-analysis of nutrition interventions during pregnancy on infant and child cognitive outcomes in developing countries found no statistically significant impact of intervention on eight domains of cognitive development, citing the need for further research.^88^ In both studies, substantial heterogeneity was observed, and tabular synthesis was needed, however as the interventions and outcomes were more specified, a meta-analysis was possible. In a 2019 Cochrane review on nutrition education and metabolic risk in children and adolescents, intervention was found to significantly impact abdominal obesity, but this outcome was not included in our review.^89^ While Leis et al. found no significant impact on all other components of metabolic syndrome, one study in our review found intervention to reduce occurrence of adolescent metabolic syndrome.

Further, despite findings of modest and absent effects in our review, there is broad consensus of the importance and effects of early nutrition on later health.^90–93^ However, it seems to be a challenge to convert these findings into impactful interventions that target dietary behaviors to influence overall health in the long-term. Therefore, more research is required to test the translation of intervention effects in population health interventions and outcomes.

### Strength and limitations

This review explored an understanding of current evidence surrounding early life nutrition interventions on long-term health. Even though, our broad research question resulted in a heterogeneity of interventions and outcomes, the synthesis of included RCTs provides an overview picture on the research work on early life nutrition interventions. This review implemented a comprehensive data search strategy and rigorous methodology and was registered in PROSPERO prior to the screening of records. The screening of registries and the adherence to the PRISMA guidelines further strengthen this review. Future research can be strengthened by targeting specific outcomes for each of the outcome domains included in this review and increasing the reporting of primary clinical endpoints in long-term follow-ups.

A meta-analysis was not possible due to the heterogeneity of data. All studies had some risk concerns across all domains and demonstrated the typical weaknesses of lifestyle intervention trials with concerns in attrition bias and missing data. There was a lack of studies in pregnancy, with only two studies starting the intervention in pregnancy but continuing into infancy. Given more primary trials, and increased homogeneity between outcomes, subgroup analyses can be insightful as to effectiveness of different types of early nutrition interventions. No conclusions can be drawn on the effectiveness of intervention type, duration, timing, or delivery method.

We decided to exclude studies that focused solely on breastfeeding as an intervention or solely on obesity, overweight and adiposity as outcomes, which could be seen as a limitation. The reason for this was that both topics are extensively studied on their own.^94–97^ Furthermore, the definition of obesity is continuously evolving, with the Canadian practice definition as “a complex chronic disease in which abnormal or excess body fat (adiposity) impairs health, increases the risk of long-term medical complications, and reduces lifespan”.^98^ Following this definition, body mass index or anthropometric measures alone are not sufficient to diagnose obesity and additional risks for disease needs to be identified, which are considered in this review as measurements of cardio-metabolic outcomes.

This review included one large-scale, long-term study with 38 included reports, which may be overrepresented and susceptible to high detection risk. The STRIP study produced numerous publications with analyses on the same dataset which may lead to multiple reports of related outcomes. Efforts were made to include only the results of the latest measurements; however, the STRIP study was the only study with such extensive follow-up that it was often the sole study reporting outcomes and may be over-weighted in the overall assessment of outcomes.

Since the completion of our search in November 2022, three new reports have been published that would have matched our inclusion criteria. One report is from the STRIP study reporting that participants in the intervention group had reduced odds of maintaining at-risk non-HDL-C from infancy to young adulthood.^99^ The STRIP study is already included in our review. The results in young adulthood only confirm the preliminary evidence of improvements of early nutritional interventions on cardio-metabolic health. The other two reports are from one study showing some favorable impact of a nutrition and exercise intervention during pregnancy on two domains of neurodevelopment and on emotion regulation in the offspring at 12 months of age.^100,101^ These studies contribute to our findings that mental health outcomes are measured in several different ways, which limits comparability between studies. This shows the need for more studies using the same outcomes and measures to assess relevance and impact.

### Recommendations for future interventions

Future research should target relevant clinical endpoints in intervention studies as well as longer follow-ups, if possible. To evaluate relevant outcomes and subsequent effects, sample sizes must be adequately planned. Additionally, it should be a part of the study to evaluate the intervention and to determine the adherence of the participants to the studied program. More homogeneity in outcome reporting is desirable with using standardized and comparable measures (e.g., establishing core outcome sets for mental health or dietary behavior) to facilitate a quantitative synthesis of findings. Future interventions should gain further insights into target periods with interventions starting during preconception or early pregnancy. Furthermore, alternative research designs such as the within cohort approach should be considered, in which community stakeholders and researchers work together on planning a long-term health strategy. This includes collecting individual health data over time to establish a cohort to be used to evaluate the effects of specific interventions. Examples are the Born in Bradford’s Better start program or the SARPHATI cohort.^102,103^

## Conclusion

There is some evidence that nutrition interventions during the first 1000 days of life increase positive and reduce unfavorable dietary behavior but have no impact on mental health. There is evidence of some preliminary positive impact on cardio-metabolic health in reducing occurrence of metabolic syndrome and improving lipid profile, with early signs of some effects being sustained into early adulthood. There is no evidence thus far on impact of nutrition interventions on respiratory health. The modest and absent evidence in our review highlights the challenge of implementing effective behavior change interventions that modify dietary behaviors in the long term and thus positively impact overall health outcomes. Innovative, long-term intervention study designs are needed for deeper insight on these findings.

## Supplementary information

Checklist

Supplementary Material I

Supplement II

Supplement III

## Data Availability

In this study only publicly available, published data was used. Further inquiries can be addressed to the corresponding author.
