# Correction: Nutrition interventions in the first 1000 days and long-term health outcomes: a systematic review

**DOI:** 10.1038/s41390-025-04344-y

**Published:** 2025-09-04

**Authors:** Anna Xu, Kathrin Guerlich, Berthold Koletzko, Veit Grote

**Affiliations:** 1 Division of Metabolic and Nutritional Medicine, Department of Pediatrics, Dr. von Hauner Children’s Hospital, LMU University Hospital, Munich, Germany; 2 German Center for Child and Adolescent Health site, Munich, Germany; 3 Nuffield Department of Primary Care Health Sciences and Department of Continuing Education, University of Oxford, Oxford, UK

Correction to: *Pediatric Research* 10.1038/s41390-025-04215-6, online 18 July 2025

The labeling of table 2 has been incorrect in the original version of this article. This has been corretced.

**Former Table 2:** Characteristics and results of included studies.

Study ID/ Report ID	Country	Intervention target / duration	Aim	Age at start	N at baseline	Outcomes reported	Cardio-metabolic health	Mental health	Diet Behavior
**Multiple Nutrition Intervention**									
**Beinert et al**.^22^ **(First food for infants)** Overby et al.^72^	Norway	Infancy 2 days	Improve child eating behavior/ parental feeding practices	4–6 mos.	110	Food Intake, Food Preference, Supplementation Lipid Profile	^a^24 mos.		^a^24 mos.
**Daniels et al. 2018** **(BLISS study)**	New Zealand	Pregnancy 10 mos.	Obesity prevention	Third trimester of pregnancy	206	Nutrient Intake			^b^12 mos.
**De Franchis et al**.^82^	Italy	Infancy 12 mos.	Improve adherence to Mediterranean diet	4–6 mos.	394	Dietary Patterns			^a^18 mos.
**Helle et al. 2019** **(Early Food for Future Health)**	Norway	Infancy 6 mos.	Improve child eating behavior/ parental feeding practices	3–5 mos.	718	Food Intake, Eating Behaviors			^c,g^24 mos.
**Hoppu et al**.^26^	Finland	Pregnancy 21 mos.	Impact on cardiovascular health	First trimester of pregnancy	256	Nutrient Intake			^d,h^12 mos. to 4 y
**Roed et al. 2021**	Norway	Infancy 6 mos.	Improve child eating behavior	11 mos.	298	Food Intake			^b^2 y
**Lifestyle Intervention**									
**Campbell et al**.^23^ **(InFANT Study)**^f^ Hesketh et al.^30^ Spence et al.^74^	Australia	Infancy 15 mos.	Obesity prevention	4 mos.	542	Dietary Patterns Food Intake			^e^18 mos. ^e^5 y
**Döring et al**.^27^ **(PRIMROSE)**^f^	Sweden	Infancy 39 mos.	Obesity prevention	5–6 mos.	1369	Food Intake			e˄4 y
**Fangupo et al**.^28^ **(POI.nz)** Taylor et al. 2018	New Zealand	Infancy 18 mos.	Improve child eating behavior/ parental feeding practices	4–6 weeks	802	Food Intake, Nutrient Intake Dietary Patterns Self-regulation abilities		^b^3.5 y	^b^2 y ^b^3.5 y
**Niinikoski et al**.^33^ **(STRIP Study)** Rask-Nissilä et al.^56^ Talvia et al.^65^ Rasanen et al.^55^ Niinikoski et al.^46^ Ruottinen et al.^60^ Niinikoski et al.^47^ Oranta et al. ^67^ Niinikoski et al.^48^ Kaseva et al. 2015, Lapinleimu et al.^42^ Nupponen et al.^49^ Lehtovirta et al.^43^ Nuotio et al.^50^ Matthews et al.^44^ Laitinen et al.^68^ Rovio et al.^70^ Pahkala et al.^69^	Finland	Infancy 26 years	Atherosclerosis and coronary heart disease prevention	7 mos.	1062	Nutrient Intake, Food Intake, Dietary Patterns, Food Knowledge Blood pressure categories Metabolic syndrome Continuous blood pressure, Systolic blood pressure, Diastolic blood pressure Cardiovascular measures Lipid Profile Biochemical Profile Cognitive Development Psychological wellbeing, School performance	e15 to 20 y e7 mos. to 15 y ^o^26 y ^o^11 y, 11 to 19 y e˄ 15 to 19 y ^a^26 y e˄ 9 to 19 y e26 y e15 to 20 y	^b^5, 26 y ^o^14, 20 y	^b^9, 26 y e˄9, 10, 19 y ^d,i^20 y ^a^20 y
**O’Sullivan et al**.^71^	Ireland	Pregnancy 6 years	Improve cognitive development	Pregnancy	233	Food Intake Cognitive development		^b^3 y	^b^3 y
**Ruggiero et al**.^83^	United States	Infancy 2.5 years	Improve child dietary behavior	10–14 days	279	Eating Behaviors			^e^2.5 y
**Van Vliet et al**.^84^	Netherlands	Infancy 12 mos.	Improve child dietary behavior	4–6 mos.	246	Food Intake			^b^2 y
**Verbestel et al**.^75^^,f^	Belgium	Infancy 1 year	Obesity prevention	9–24 mos.	203	Food Intake			^b^9–24 mos.
**Wen et al**.^77^	Australia	Pregnancy 26 mos.	Obesity prevention	Third trimester of pregnancy	667	Food Intake Quality of Life, Physical/ Emotional/Social/School Functioning		^b^5 y	^b^5 y
**Wen et al**.^79^	Australia	Pregnancy, infancy 2 years	Improve child eating behavior/ parental feeding practices	Third trimester of pregnancy	1155	Food Intake			^b^2 y
**Nutrition Education**									
**Childs et al**.^24^	United Kingdom	Infancy 18 mos.	Anemia prevention	Birth	1000	Dietary Patterns			^b^18 mos.
**Daniels et al**.^25^ **(NOURISH RCT)** Magarey et al.^32^	Australia	Infancy 18 mos.	Improve child eating behavior/ parental feeding practices	2–7 mos.	698	Food Intake, Eating Behavior, Food Preferences, Dietary Patterns			e24 mos. ^b^24 mos. to 5 y
**Watt et al**.^76^	United Kingdom	Infancy 12 mos.	Improve child eating behavior/ parental feeding practices	10 weeks	312	Food Intake, Nutrient Intake			^d^^,^^j^18 mos.
**Wozniak et al**.^80^ Wozniak et al.^80^	Poland	Infancy 12 mos.	Impact on child iron and metabolic status	under 8 weeks	203	Nutrient Intake Lipid Profile	^e^1 y		^e^˄1 y

*mos* months, *y* year.

^a^Increased favorable behavior.

^b^No difference.

^c^Favored control.

^d^Conflicting significant results (had both favored control and intervention).

^e^Decreased unfavorable behavior.

^f^Cluster RCT.

^g^Favored control group for emotional overeating score and food responsiveness score.

^h^Favored control group for mean vitamin C intake (95% CI), I: 66 mg/day (62–69), C: 75 mg/day (68–81), treatment effect 9.2 (1.6–16.9), *p* = 0.017, however both groups had over recommended daily intake for vitamin C.

^i^Favored control group for decreasing sodium intake, ß-coeff (95%-CI): 55.00, (24.40–85.60 mg/day), *p* < 0.001.

^j^Favored control for decreased consumption of chips and other potatoes.

Intervention Types: (1) nutrition education (2) meal planning (theoretical/practical), (3) complementary food, (4) supplements, (5) multiple—combination of 1–4, (6) lifestyle interventions—combination of 1–4 with other lifestyle related interventions (e.g., promoting physical activity, parenting education, healthy behaviors). All controls used were standard care unless indicated.

**Corrected Table 2:** Characteristics and results of included studies

Study ID/Report ID	Country	Intervention target / duration	Aim	Age at start	N at baseline	Outcomes reported	Cardio-metabolic health	Mental health	Diet Behavior
**Multiple Nutrition Intervention**									
**Beinert et al**.^22^ **(First food for infants)** Overby et al.^72^	Norway	Infancy 2 days	Improve child eating behavior/ parental feeding practices	4–6 mos.	110	Food Intake, Food Preference, Supplementation Lipid Profile	^a^24 mos.		^a^24 mos.
**Daniels et al**.^26^ **(BLISS study)**	New Zealand	Pregnancy 10 mos.	Obesity prevention	Third trimester of pregnancy	206	Nutrient Intake			^b^12 mos.
**De Franchis et al**.^82^	Italy	Infancy 12 mos.	Improve adherence to Mediterranean diet	4–6 mos.	394	Dietary Patterns			^a^18 mos.
**Helle et al**.^29^ **(Early Food for Future Health)**	Norway	Infancy 6 mos.	Improve child eating behavior/ parental feeding practices	3–5 mos.	718	Food Intake, Eating Behaviors			^c,g^24 mos.
**Hoppu et al**.^31^	Finland	Pregnancy 21 mos.	Impact on cardiovascular health	First trimester of pregnancy	256	Nutrient Intake			^d,h^12 mos. to 4 y
**Roed et al**.^78^	Norway	Infancy 6 mos.	Improve child eating behavior	11 mos.	298	Food Intake			^b^2 y
**Lifestyle Intervention**									
**Campbell et al**.^23^ **(InFANT Study)**^f^ Hesketh et al.^30^, Spence et al.^74^	Australia	Infancy 15 mos.	Obesity prevention	4 mos.	542	Dietary Patterns Food Intake			^e^18 mos. ^e^5 y
**Döring et al**.^27^ **(PRIMROSE)**^f^	Sweden	Infancy 39 mos.	Obesity prevention	5–6 mos.	1369	Food Intake			^a,^^e^4 y
**Fangupo et al**.^28^ **(POI.nz)** Taylor et al.^73^	New Zealand	Infancy 18 mos.	Improve child eating behavior/ parental feeding practices	4–6 weeks	802	Food Intake, Nutrient Intake Dietary Patterns Self-regulation abilities		^b^3.5 y	^b^2 y ^b^3.5 y
**Niinikoski et al**.^33^ **(STRIP Study)** Rask-Nissilä et al.^56^ Talvia et al.^65^ Räsänen et al.^55^ Niinikoski et al.^46^ Ruottinen et al.^60^ Niinikoski et al.^47^ Oranta et al.^67^ Niinikoski et al.^48^ Kaseva et al.^41^ Lapinleimu et al.^42^ Nupponen et al.^49^ Lehtovirta et al.^43^ Nuotio et al.^50^ Matthews et al.^44^ Laitinen et al.^68^ Rovio et al.^70^ Pahkala et al.^69^	Finland	Infancy 26 years	Atherosclerosis and coronary heart disease prevention	7 mos.	1062	Nutrient Intake, Food Intake, Dietary Patterns, Food Knowledge Blood pressure categories Metabolic syndrome Continuous blood pressure, Systolic blood pressure, Diastolic blood pressure Cardiovascular measures Lipid Profile Biochemical Profile Cognitive Development Psychological wellbeing, School performance	^e^15 to 20 y ^e^7 mos. to 15 y ^b^26 y ^b^11 y, 11 to 19 y ^a,e^15 to 19 y ^a^26 y ^a,e^9 to 19 y ^e^26 y ^e^15 to 20 y	^b^5, 26 y ^b^14, 20 y	^b^9, 26 y ^a,e^9, 10, 19 y ^d,i^20 y ^a^20 y
**O’Sullivan et al**.^71^	Ireland	Pregnancy 6 years	Improve cognitive Development	Pregnancy	233	Food Intake Cognitive Development		^b^3 y	^b^3 y
**Ruggiero et al**.^83^	United States	Infancy 2.5 years	Improve child dietary behaviour	10–14 days	279	Eating Behaviours			^e^2.5 y
**Van Vliet et al**.^84^	Netherlands	Infancy 12 mos.	Improve child dietary behaviour	4–6 mos.	246	Food Intake			^b^2 y
**Verbestel et al**.^75^^,*f*^	Belgium	Infancy 1 year	Obesity prevention	9–24 mos.	203	Food Intake			^b^9 to 24 mos.
**Wen et al**.^77^	Australia	Pregnancy 26 mos.	Obesity prevention	Third trimester of pregnancy	667	Food Intake Quality of Life, Physical/Emotional/Social/School Functioning		^b^5 y	^b^5 y
**Wen et al**.^79^	Australia	Pregnancy, infancy 2 years	Improve child eating behaviour/ parental feeding practices	Third trimester of pregnancy	1155	Food Intake			^b^2 y
**Nutrition Education**									
**Childs et al**.^24^	United Kingdom	Infancy 18 mos.	Anaemia prevention	Birth	1000	Dietary Patterns			^b^18 mos.
**Daniels et al**.^25^ **(NOURISH RCT)** Magarey et al.^32^	Australia	Infancy 18 mos.	Improve child eating behaviour/ parental feeding practices	2–7 mos.	698	Food Intake, Eating Behaviour, Food Preferences, Dietary Patterns			^e^24 mos. ^b^24 mos. to 5 y
**Watt et al**.^76^	United Kingdom	Infancy 12 mos.	Improve child eating behaviour/ parental feeding practices	10 weeks	312	Food Intake, Nutrient Intake			^d,j^18 mos.
**Wozniak et al**.^80^ Wozniak et al.^81^	Poland	Infancy 12 mos.	Impact on child iron and metabolic status	Under 8 weeks	203	Nutrient Intake Lipid Profile	^e^1 y		^a,e^1 y

*mos* months, *y* year

^a^Increased favorable behavior

^b^No difference

^c^Favored control

^d^Conflicting significant results (had both favored control and intervention)

^e^Decreased unfavorable behavior

^f^Cluster RCT

^g^Favored control group for emotional overeating score and food responsiveness score.

^h^Favored control group for mean vitamin C intake (95% CI), I: 66 mg/day (62–69), C: 75 mg/day (68–81), treatment effect (9.2 (1.6 to 16.9), p = 0.017, however both groups had over recommended daily intake for vitamin C.

^i^Favored control group for decreasing sodium intake, ß-coeff (95%-CI): 55.00, (24.40 to 85.60 mg/day), p < 0.001.

^j^Favored control for decreased consumption of chips and other potatoes.

Intervention Types: 1) nutrition education 2) meal planning (theoretical/practical), 3) complementary food, 4) supplements, 5) multiple - combination of 1–4, 6) lifestyle interventions - combination of 1–4 with other lifestyle related interventions (e.g., promoting physical activity, parenting education, healthy behaviors). All controls used were standard care unless indicated.

The original article has been corrected.

