# Supplementary material for: Nutrition interventions in the first 1000 days and long-term health outcomes: a systematic review
Source: Pediatr Res. 2025 Jul 18;98(6):2023–34. doi: 10.1038/s41390-025-04215-6 (PMC12811146; doi:10.1038/s41390-025-04215-6)
Supplement: Supplementary file 2 — Supplementary Material I [file 41390_2025_4215_MOESM2_ESM.pdf]

Supplementary Material I

Nutrition interventions in the first 1000 days and long-term health outcomes: a systematic review

Anna Xu, Kathrin Guerlich, Berthold Koletzko*, Veit Grote

***Corresponding author:**
Berthold Koletzko, MD, PhD, Division of Metabolic and Nutritional Medicine, Department of Pediatrics, Dr. von Hauner Children's Hospital, LMU University Hospital, LMU Munich, Lindwurmstr. 4, D-80337 München. Email: office.koletzko@med.uni-muenchen.de. Phone: +49 (0)89 4400 53967. Fax: +49 (0)89 4400 57742.

Table S1: Full outcomes

Cardio-metabolic outcomes:
- Primary outcomes:
  - Cardiovascular disease (e.g., hypertension)
  - Cardiovascular measures (e.g., blood pressure)
  - Metabolic disease (e.g., diabetes mellitus)
- Secondary outcomes:
  - Serum Markers
    - Inflammatory measurements (e.g., CRP)
    - Lipid measures (e.g., fatty acids, total cholesterol, triglycerides)
    - Blood glucose (e.g., glucose, HbA1c, insulin)
  - Other cardiovascular measures (e.g., carotid intima media thickness)

Respiratory outcomes:
- Primary outcomes:
  - Respiratory diseases
    - Wheezing
    - Asthma
    - COPD
- Secondary outcomes:
  - Measure of lung function (e.g., FEV1, FVC, bronchial hyperresponsiveness, fractional exhaled nitric oxide)

Mental Health outcomes:
- Primary outcomes:
  - Mental health diagnoses
    - ADHD (Attention Deficit Hyperactivity Disorder)
    - ASD (Autism-Spectrum Disorder)
    - Internalizing and externalizing behavior
  - Self-assessment/reporting of mental health status
- Secondary outcomes:
  - Psychomotor development (e.g., gross motor and fine motor)
  - Executive Function (e.g., working memory)
  - Intelligence (nonverbal) (e.g., IQ)
  - Language

Child diet behaviour outcomes:
- Primary outcomes:
  - Dietary intake (e.g., portions of fruits, vegetables, sodium intake)
  - Diet quality (e.g., variation of intake)
  - Nutrition knowledge

Table S2: Full-text publications excluded with reasons

[References]	Reason for exclusion
[1-29]	Non-eligible study population (n = 29)
[30-45]	Not outcome of interest (n = 16)
[46-60]	Study protocol (n = 15)
[61-69]	Duplicate (n = 9)
[70-77]	Short follow-up <12 months (n = 8)
[78-83]	Not-suitable intervention (n = 6)
[84-88]	Non-eligible study design (n = 5)
[89-91]	Conferences - irrelevant topics (n = 3)
[92, 93]	Study ongoing (n = 2)
[94, 95]	Not-suitable setting (n = 2)
[96]	Not-suitable control group (n = 1)

References

Non-eligible study population (n = 29)

1. Nct. Childhood Obesity Microbiome Study. https://clinicaltrialsgov/show/NCT04770311. 2021.
2. Arvidsson, L., Bogl, L. H., Eiben, G., Hebestreit, A., Nagy, P., Tornaritis, M., Moreno, L. A., Siani, A., Veidebaum, T., de Henauw, S., & et al. (2015). Fat, sugar and water intakes among families from the IDEFICS intervention and control groups: first observations from I.Family. *Obesity reviews, 16*, 127 ‐ 137. doi:10.1111/obr.12325
3. Banerjee, T., & Nayak, A. (2017). Believe it or not: Health education works. *Obes Res Clin Pract, 12*(1), 116-124. doi:10.1016/j.orcp.2017.09.001
4. Bannon, K., & Schwartz, M. B. (2006). Impact of nutrition messages on children's food choice: pilot study. *Appetite, 46*(2), 124-129. doi:10.1016/j.appet.2005.10.009
5. Bayer, O., von Kries, R., Strauss, A., Mitschek, C., Toschke, A. M., Hose, A., & Koletzko, B. V. (2009). Short- and mid-term effects of a setting based prevention program to reduce obesity risk factors in children: a cluster-randomized trial. *Clin Nutr, 28*(2), 122-128. doi:10.1016/j.clnu.2009.01.001
6. Beckerman, J. P., Aftosmes-Tobio, A., Kitos, N., Jurkowski, J. M., Lansburg, K., Kazik, C., Gavarkovs, A., Vigilante, A., Kalyoncu, B., Figueroa, R., Klabunde, R., Barouch, R., Haneuse, S., Taveras, E., & Davison, K. K. (2019). Communities for healthy living (CHL) - A family-centered childhood obesity prevention program integrated into Head Start services: Study protocol for a pragmatic cluster randomized trial. *Contemporary Clinical Trials, 78*, 34-45. doi:http://dx.doi.org/10.1016/j.cct.2019.01.002
7. Bourcier, E., Bowen, D. J., Meischke, H., & Moinpour, C. (2003). Evaluation of strategies used by family food preparers to influence healthy eating. *Appetite, 41*(3), 265-272. doi:10.1016/s0195-6663(03)00104-1
8. Davis, A. M., Stough, C. O., Black, W. R., Dean, K., Sampilo, M., Simpson, S., & Landrum, Y. (2016). Outcomes of a weight management program conjointly addressing parent and child health. *Children's Health Care, 45*(2), 227-240. doi:http://dx.doi.org/10.1080/02739615.2014.979923
9. Dumas, A. A., Lemieux, S., Lapointe, A., Provencher, V., Robitaille, J., & Desroches, S. (2017). Development of an Evidence-Informed Blog to Promote Healthy Eating Among Mothers: Use of the Intervention Mapping Protocol. *JMIR Res Protoc, 6*(5), e92. doi:10.2196/resprot.7147

10. Gratton, L., Povey, R., & Clark-Carter, D. (2007). Promoting children's fruit and vegetable consumption: interventions using the Theory of Planned Behaviour as a framework. *British journal of health psychology, 12*(Pt 4), 639 - 650. doi:10.1348/135910706X171504
11. Hoffman, J. A., Thompson, D. R., Franko, D. L., Power, T. J., Leff, S. S., & Stallings, V. A. (2011). Decaying behavioral effects in a randomized, multi-year fruit and vegetable intake intervention. *Prev Med, 52*(5), 370-375. doi:10.1016/j.ypmed.2011.02.013
12. Hunsaker, S. L., & Jensen, C. D. (2017). Effectiveness of a Parent Health Report in Increasing Fruit and Vegetable Consumption Among Preschoolers and Kindergarteners. *J Nutr Educ Behav, 49*(5), 380-386.e381. doi:10.1016/j.jneb.2017.01.002
13. Knowlden, A., & Sharma, M. (2016). One-Year Efficacy Testing of Enabling Mothers to Prevent Pediatric Obesity Through Web-Based Education and Reciprocal Determinism (EMPOWER) Randomized Control Trial. *Health Education & Behavior, 43*(1), 94-106. doi:10.1177/1090198115596737
14. Knowlden, A. P., & Conrad, E. (2018). Two-Year Outcomes of the Enabling Mothers to Prevent Pediatric Obesity Through Web-Based Education and Reciprocal Determinism (EMPOWER) Randomized Control Trial. *Health Education & Behavior, 45*(2), 262-276. doi:10.1177/1090198117732604
15. Koletzko, B., Brands, B., Chourdakis, M., Cramer, S., Grote, V., Hellmuth, C., Kirchberg, F., Prell, C., Rzehak, P., Uhl, O., & Weber, M. (2014). The Power of Programming and the EarlyNutrition project: opportunities for health promotion by nutrition during the first thousand days of life and beyond. *Ann Nutr Metab, 64*(3-4), 187-196. doi:10.1159/000365017
16. Kolu, P., Raitanen, J., Puhkala, J., Tuominen, P., Husu, P., & Luoto, R. (2016). Effectiveness and Cost-Effectiveness of a Cluster-Randomized Prenatal Lifestyle Counseling Trial: A Seven-Year Follow-Up. *PLoS One, 11*(12), e0167759. doi:10.1371/journal.pone.0167759
17. Ling, J., Zahry, N. R., & Robbins, L. B. (2019). Dose-Response Relationship in a Healthy Habits Study for Head Start Preschoolers. *Nurs Res, 68*(4), 329-335. doi:10.1097/nnr.0000000000000345
18. Miller, A. L., Horodynski, M. A., Herb, H. E. B., Peterson, K. E., Contreras, D., Kaciroti, N., Staples-Watson, J., & Lumeng, J. C. (2012). Enhancing self-regulation as a strategy for obesity prevention in Head Start preschoolers: the growing healthy study. *BMC Public Health, 12*(1), 1040-1040. doi:10.1186/1471-2458-12-1040
19. Natale, R. A., Messiah, S. E., Asfour, L. S., Uhlhorn, S. B., Englebert, N. E., & Arheart, K. L. (2017). Obesity Prevention Program in Childcare Centers: Two-Year Follow-Up. *Am J Health Promot, 31*(6), 502-510. doi:10.1177/0890117116661156
20. Paineau, D., Beaufils, F., Boulier, A., Cassuto, D. A., Chwalow, J., Combris, P., Couet, C., Jouret, B., Lafay, L., Laville, M., & et al. (2010). The cumulative effect of small dietary changes may significantly improve nutritional intakes in free-living children and adults. *European journal of clinical nutrition, 64*(8), 782 - 791. doi:10.1038/ejcn.2010.78
21. Pellanda, L. C., Minossi, V., Borges, R. F., & Hattge Jr, S. P. (2015). The happy heart educational program randomized clinical trial: Benefits for children with cardiovascular risk factors and their caregivers are sustained after a year of follow-up. *European Heart Journal, 36*(SUPPL. 1), 114. doi:http://dx.doi.org/10.1093/eurheartj/ehv398
22. Perry, R. A., Daniels, L., Baur, L. A., & Magarey, A. (2018). Impact of a 6-month family-based weight management programme on child food and activity behaviours:

short-term and long-term outcomes of the PEACH intervention. *Pediatr Obes, 13*(11), 744-751. doi:10.1111/ijpo.12460

23. Reinaerts, E., Crutzen, R., Candel, M., De Vries, N. K., & De Nooijer, J. (2008). Increasing fruit and vegetable intake among children: Comparing long-term effects of a free distribution and a multicomponent program. *Health Education Research, 23*(6), 987-996. doi:http://dx.doi.org/10.1093/her/cyn027
24. Scheiwe, A., Hardy, R., & Watt, R. G. (2010). Four-year follow-up of a randomized controlled trial of a social support intervention on infant feeding practices. *Matern Child Nutr, 6*(4), 328-337. doi:10.1111/j.1740-8709.2009.00231.x
25. Sharps, M., & Robinson, E. (2016). Encouraging children to eat more fruit and vegetables: health vs. descriptive social norm-based messages. *Appetite, 100*, 18 - 25. doi:10.1016/j.appet.2016.01.031
26. Walton, K., Filion, A. J., Darlington, G., Morrongiello, B., & Haines, J. (2015). Parents and tots together: adaptation of a family-based obesity prevention intervention to the Canadian context. *Canadian journal of diabetes., 39*, S72 - S73. Retrieved from https://www.cochranelibrary.com/central/doi/10.1002/central/CN-01129845/full
27. Wen, L. M., Rissel, C., Xu, H., Taki, S., Smith, W., Bedford, K., Hayes, A. J., Phongsavan, P., Simpson, J. M., Shaw, M. J., Moreton, R., & Baur, L. A. (2019). Linking two randomised controlled trials for Healthy Beginnings(c): optimising early obesity prevention programs for children under 3 years. *BMC Public Health, 19*(1), 739. doi:10.1186/s12889-019-7058-9
28. Williams, P. A., Cates, S. C., Blitstein, J. L., Hersey, J. C., Kosa, K. M., Long, V. A., Singh, A., & Berman, D. (2015). Evaluating the Impact of Six Supplemental Nutrition Assistance Program Education Interventions on Children's At-Home Diets. *Health Educ Behav, 42*(3), 329-338. doi:10.1177/1090198114558589
29. Wolfenden, L., Wyse, R., Campbell, E., Brennan, L., Campbell, K. J., Fletcher, A., Wiggers, J., Bowman, J., & Heard, T. R. (2014). Randomized controlled trial of a telephone-based intervention for child fruit and vegetable intake: long-term follow-up. *Am J Clin Nutr, 99*(3), 543-550. doi:10.3945/ajcn.113.071738

Not outcome of interest (n = 16)

30. Himberg-Sundet A, Kristiansen AL, Frost Andersen L, Bjelland M, Lien N. Effects of a kindergarten intervention on vegetables served and staff's food-related practices: results of a cluster randomised controlled trial - the BRA study. Public health nutrition. 2020;23(6):1117-26.
31. Laitinen TT, Nuotio J, Niinikoski H, Juonala M, Rovio SP, Viikari JSA, et al. Attainment of Targets of the 20-Year Infancy-Onset Dietary Intervention and Blood Pressure Across Childhood and Young Adulthood: The Special Turku Coronary Risk Factor Intervention Project (STRIP). Hypertension (0194911X). 2020;76(5):1572-9.
32. Wen LM, Rissel C, Xu H, Taki S, Buchanan L, Bedford K, et al. Effects of Telephone and Short Message Service Support on Infant Feeding Practices, "Tummy Time," and Screen Time at 6 and 12 Months of Child Age: a 3-Group Randomized Clinical Trial. JAMA pediatrics. 2020;174(7):657-64.
33. Prevention of Obesity in Toddlers (PROBIT) Trial. In.
34. Adamo, K. B., Ferraro, Z. M., Goldfield, G., Keely, E., Stacey, D., Hadjiyannakis, S., Jean-Philippe, S., Walker, M., & Barrowman, N. J. (2013). The Maternal Obesity Management (MOM) Trial Protocol: a lifestyle intervention during pregnancy to minimize downstream obesity. *Contemp Clin Trials, 35*(1), 87-96. doi:10.1016/j.cct.2013.02.010
35. Cloutier, M. M., Wiley, J. F., Kuo, C. L., Cornelius, T., Wang, Z., & Gorin, A. A.

(2018). Outcomes of an early childhood obesity prevention program in a low-income community: a pilot, randomized trial. *Pediatr Obes, 13*(11), 677-685. doi:10.1111/ijpo.12458
36. Haire-Joshu, D., Brownson, R. C., Nanney, M. S., Houston, C., Steger-May, K., Schechtman, K., & Auslander, W. (2003). Improving dietary behavior in African Americans: the Parents As Teachers High 5, Low Fat Program. *Preventive medicine, 36*(6), 684 - 691. doi:10.1016/s0091-7435(03)00053-7
37. Havas, S., Damron, D., Treiman, K., Anliker, J., Langenberg, P., Hammad, T. A., Ballesteros, M., Freimuth, V., & Feldman, R. (1997). The Maryland WIC 5 A Day Promotion Program pilot study: rationale, results, and lessons learned. *Journal of nutrition education, 29*(6), 343 - 350. Retrieved from https://www.cochranelibrary.com/central/doi/10.1002/central/CN-00635579/full
38. Isrctn. (2003). Randomised trial of pre-pregnancy information and counselling in inner urban Melbourne. *http://www.who.int/trialsearch/Trial2.aspx?TrialID=ISRCTN20165116*. Retrieved from https://www.cochranelibrary.com/central/doi/10.1002/central/CN-01874325/full
39. Jancey, J. M., Dos Remedios Monteiro, S. M., Dhaliwal, S. S., Howat, P. A., Burns, S., Hills, A. P., & Anderson, A. S. (2014). Dietary outcomes of a community based intervention for mothers of young children: a randomised controlled trial. *Int J Behav Nutr Phys Act, 11*, 120. doi:10.1186/s12966-014-0120-1
40. Katzow, M., Mendelsohn, A., Gross, R., & Messito, M. J. (2019). NP20 Starting Early: Expansion of a Primary Care-Based Early Child Obesity Prevention Program...Society for Nutrition Education and Behavior 52nd Annual Conference, Nutrition Education: Rooted in Food, July 27-30, 2019, Orlando, Florida. *Journal of Nutrition Education & Behavior, 51*, S19-S19. doi:10.1016/j.jneb.2019.05.344
41. Li Ming, W., Chris, R., Baur, L. A., Hayes, A. J., Huilan, X., Whelan, A., Myna, H., Shaw, M., Phongsavan, P., Wen, L. M., Rissel, C., Xu, H., & Hua, M. (2017). A 3-Arm randomised controlled trial of Communicating Healthy Beginnings Advice by Telephone (CHAT) to mothers with infants to prevent childhood obesity. *BMC Public Health, 17*(1), 1-10. doi:10.1186/s12889-016-4005-x
42. Mustila, T., Raitanen, J., Keskinen, P., Saari, A., & Luoto, R. (2012). Lifestyle counseling during pregnancy and offspring weight development until four years of age: follow-up study of a controlled trial. *J Negat Results Biomed, 11*, 11. doi:10.1186/1477-5751-11-11
43. Sanders, L. M., Perrin, E. M., Yin, H. S., Bronaugh, A., & Rothman, R. L. (2014). "Greenlight study": a controlled trial of low-literacy, early childhood obesity prevention. *Pediatrics, 133*(6), e1724-1737. doi:10.1542/peds.2013-3867
44. Savage, J. S., Hohman, E. E., Marini, M. E., Shelly, A., Paul, I. M., & Birch, L. L. (2018). INSIGHT responsive parenting intervention and infant feeding practices: randomized clinical trial. *Int J Behav Nutr Phys Act, 15*(1), 64. doi:10.1186/s12966-018-0700-6
45. Tarmi-Mattsson, M., Keskinen, S., Korhonen, T. T., Lapinleimu, H., Tuominen, J., Niinikoski, H., Viikari, J., Ronnemaa, T., Valimaki, I., & Simell, O. (1997). Behavior of 3-year old children in a prospective randomized trial of reduced saturated fat and cholesterol diet since infancy: the STRIP baby project. *Int J Behav Med, 4*(4), 310-322. doi:10.1207/s15327558ijbm0404_4

Study protocol (n = 15)

46. Nct. Cardiovascular Risk Reduction-Diet in Pregnancy - Follow up 20 Years After the "CARRDIP"-Trial. https://clinicaltrialsgov/show/NCT05030922. 2021.

47. Nl. Prevention of asthma and respiratory symptoms in young children with a nutritional intervention. http://wwwwhoint/trialsearch/Trial2aspx?TrialID=NL8752. 2020.
48. Pactr. Effectiveness of Optimal Child Food Formulation and Nutrition Education in Improving Nutritional and Iron Status of Infant and Young children. https://trialsearchwhoint/Trial2aspx?TrialID=PACTR202203713065634. 2022.
49. Campbell, K., Hesketh, K., Crawford, D., Salmon, J., Ball, K., & McCallum, Z. (2008). The Infant Feeding Activity and Nutrition Trial (INFANT) an early intervention to prevent childhood obesity: cluster-randomised controlled trial. *BMC Public Health, 8*, 103. doi:10.1186/1471-2458-8-103
50. Campbell, K., McNaughton, S., Lioret, S., Crawford, D., Salmon, J., Ball, K., McCallum, Z., & Hesketh, K. (2011). The Melbourne InFANT Program positively impacts very young children's lifestyle patterns. *Obesity Research and Clinical Practice, 5*(SUPPL. 1), S4-S5. doi:http://dx.doi.org/10.1016/j.orcp.2011.08.059
51. Campbell, K. J., Hesketh, K. D., McNaughton, S. A., Ball, K., McCallum, Z., Lynch, J., & Crawford, D. A. (2016). The extended Infant Feeding, Activity and Nutrition Trial (InFANT Extend) Program: a cluster-randomized controlled trial of an early intervention to prevent childhood obesity. *BMC Public Health, 16*, 166. doi:10.1186/s12889-016-2836-0
52. Daniels, L. A., Magarey, A., Battistutta, D., Nicholson, J. M., Farrell, A., Davidson, G., & Cleghorn, G. (2009). The NOURISH randomised control trial: positive feeding practices and food preferences in early childhood - a primary prevention program for childhood obesity. *BMC Public Health, 9*, 387. doi:10.1186/1471-2458-9-387
53. Doering, N., Hansson, L. M., Andersson, E. S., Bohman, B., Westin, M., Magnusson, M., Larsson, C., Sundblom, E., Willmer, M., Blennow, M., Heitmann, B. L., Forsberg, L., Wallin, S., Tynelius, P., Ghaderi, A., & Rasmussen, F. (2014). Primary prevention of childhood obesity through counselling sessions at Swedish child health centres: design, methods and baseline sample characteristics of the PRIMROSE cluster-randomised trial. *BMC Public Health, 14*, 335. doi:10.1186/1471-2458-14-335
54. Helle, C., Hillesund, E. R., Omholt, M. L., & Overby, N. C. (2017). Early food for future health: a randomized controlled trial evaluating the effect of an eHealth intervention aiming to promote healthy food habits from early childhood. *BMC Public Health, 17*(1), 729. doi:10.1186/s12889-017-4731-8
55. Paul, I. M., Williams, J. S., Anzman-Frasca, S., Beiler, J. S., Makova, K. D., Marini, M. E., Hess, L. B., Rzucidlo, S. E., Verdiglione, N., Mindell, J. A., & Birch, L. L. (2014). The Intervention Nurses Start Infants Growing on Healthy Trajectories (INSIGHT) study. *BMC Pediatr, 14*, 184. doi:10.1186/1471-2431-14-184
56. Taylor, B. J., Heath, A.-L. M., Galland, B. C., Gray, A. R., Lawrence, J. A., Sayers, R. M., Dale, K., Coppell, K. J., & Taylor, R. W. (2011). Prevention of Overweight in Infancy (POI.nz) study: a randomised controlled trial of sleep, food and activity interventions for preventing overweight from birth. *BMC Public Health, 11*(1), 942-942. doi:10.1186/1471-2458-11-942
57. Thomson, J. L., Tussing-Humphreys, L. M., & Goodman, M. H. (2014). Delta Healthy Sprouts: a randomized comparative effectiveness trial to promote maternal weight control and reduce childhood obesity in the Mississippi Delta. *Contemp Clin Trials, 38*(1), 82-91. doi:10.1016/j.cct.2014.03.004
58. Van Der Veek, S. M. C., De Graaf, C., De Vries, J. H. M., Jager, G., Vereijken, C. M. J. L., Weenen, H., Van Winden, N., Van Vliet, M. S., Schultink, J. M., De Wild, V. W. T., Janssen, S., & Mesman, J. (2019). Baby's first bites: A randomized controlled trial to assess the effects of vegetable-exposure and sensitive feeding on vegetable acceptance, eating behavior and weight gain in infants and toddlers. *BMC Pediatrics,*

19(1), 266. doi:http://dx.doi.org/10.1186/s12887-019-1627-z
59. Wen, L. M., Baur, L. A., Rissel, C., Flood, V., Simpson, J. M., Hayes, A., Hardy, L. L., & Wardle, K. Healthy Beginnings Trial Phase 2 study: Follow-up and cost-effectiveness analysis. *Contemporary Clinical Trials*. doi:http://dx.doi.org/10.1016/j.cct.2011.11.008
60. Helland, S. H., Bere, E., & Overby, N. C. (2016). Study protocol for a multi-component kindergarten-based intervention to promote healthy diets in toddlers: a cluster randomized trial. *BMC Public Health, 16*, 273. doi:10.1186/s12889-016-2952-x

Duplicate (n = 9)

61. Hesketh KD, Salmon J, McNaughton SA, Crawford D, Abbott G, Cameron AJ, et al. Long-term outcomes (2 and 3.5 years post-intervention) of the INFANT early childhood intervention to improve health behaviors and reduce obesity: cluster randomised controlled trial follow-up. International Journal of Behavioral Nutrition & Physical Activity. 2020;17(1):1-10. https://doi.org/10.1186/s12966-020-00994-9
62. First food for infants. Retrieved from abstract available: http://www.isrctn.com/ISRCTN45864056?q=Øverby&filters=trialStatus:Completed&sort=&offset=1&totalResults=1&page=1&pageSize=10&searchType=basic-search
63. PRIMROSE: Primary prevention of childhood obesity at child health centers. Retrieved from paper accessible from: https://pubmed.ncbi.nlm.nih.gov/24717011/
64. Fangupo et al. 2015 *American Journal of Clinical Nutrition, 102*(3), 704-712. doi:http://dx.doi.org/10.3945/ajcn.115.111823 Fangupo, L. J., Heath, A.-L. M., Williams, S. M., Somerville, M. R., Lawrence, J. A., Gray, A. R., Taylor, B. J., Mills, V. C., Watson, E. O., Galland, B. C., Sayers, R. M., Hanna, M. B., & Taylor, R. W. (2015). Impact of an early-life intervention on the nutrition behaviors of 2-y-old children: a randomized controlled trial. *American Journal of Clinical Nutrition, 102*(3), 704-712. doi:10.3945/ajcn.115.111823
65. Gibson, S., & Gibson, S. (2007). Peer-led approaches to dietary change: report of the Food Standards Agency seminar held on 19 July 2006. *Public Health Nutrition, 10*(10), 980-988. Retrieved from http://search.ebscohost.com/login.aspx?direct=true&db=cin20&AN=105830843&site=ehost-live&authtype=ip,uid
66. Kaitosaari, T., Rönnemaa, T., Viikari, J., Langström, H., Niinikoski, H., Hakanen, M., & Simell, O. (2005). The effect of infancy-onset dietary intervention on serum lipoproteins and growth of children in a prospective randomized STRIP study. *Suomen lääkärilehti, 60*(1), 21 - 26. Retrieved from https://www.cochranelibrary.com/central/doi/10.1002/central/CN-00617071/full
67. Kohler, S., Sichert-Hellert, W., & Kersting, M. (2007). Effectiveness of preventive nutritional counseling during the 1st year of life. A randomized controlled intervention trial. *Monatsschrift fur Kinderheilkunde, 155*(1), 53-60. doi:http://dx.doi.org/10.1007/s00112-005-1252-9
68. Lioret, S., Campbell, K., McNaughton, S., Crawford, D., Salmon, J., Ball, K., McCallum, Z., Gerner, B., Spence, A., Cameron, A., Hnatiuk, J., Ukoumunne, O., Gold, L., & Hesketh, K. (2012). Parent focused intervention impacts obesity risk behaviours in infants: Results of the melbourne infant program cluster-randomised controlled trial. *Obesity Facts, 5*(SUPPL. 1), 33. doi:http://dx.doi.org/10.1159/000188329
69. Talvia, S., Lagstrom, H., Ronnemaa, T., Viikari, J., & Simell, O. (2002). Impact of infancy-onset dietary intervention aimed at fat modification on vitamin and mineral intake in 5- to 10-year old children, a prospective randomised trial. *Atherosclerosis, 3*(2 Suppl), 215. Retrieved from

https://www.cochranelibrary.com/central/doi/10.1002/central/CN-00593599/full

Short follow-up <12 months (n = 8)

70. Hohman, E. E., Savage, J. S., Paul, I. M., & Birch, L. L. (2016). INSIGHT study parenting intervention to prevent childhood obesity improves patterns of dietary exposures in infants. *FASEB Journal, 30*(Meeting Abstracts). Retrieved from http://www.fasebj.org/content/30/1_Supplement/295.2.abstract?sid=b176f061-91a5-46d9-95b6-df764050b0a4
71. Koehler, S., Sichert-Hellert, W., & Kersting, M. (2007). Measuring the effects of nutritional counseling on total infant diet in a randomized controlled intervention trial. *J Pediatr Gastroenterol Nutr, 45*(1), 106-113. doi:10.1097/MPG.0b013e3180331e2a
72. Lapinleimu, H., Viikari, J., Jokinen, E., Salo, P., Routi, T., Leino, A., Rönnemaa, T., Seppänen, R., Välimäki, I., & Simell, O. (1995). Prospective randomised trial in 1062 infants of diet low in saturated fat and cholesterol. *Lancet, 345 North American Edition*(8948), 471-476. Retrieved from http://search.ebscohost.com/login.aspx?direct=true&db=cin20&AN=107273874&site=ehost-live&authtype=ip,uid
73. Lapinleimu, H., Jokinen, E., Myyrinmaa, A., Viikari, J., Ronnemaa, T., Valimaki, I., Linko, L., & Simell, O. (1994). Individualized dietary counselling of families: serum cholesterol concentration and growth of children aged 7-13 months. *Acta Paediatr, 83*(4), 383-388. doi:10.1111/j.1651-2227.1994.tb18124.x
74. Nct. (2015). OTIS - Optimized Complementary Feeding Study. *https://clinicaltrials.gov/show/NCT02634749*. Retrieved from https://www.cochranelibrary.com/central/doi/10.1002/central/CN-02044757/full
75. Roed, M., Hillesund, E. R., Vik, F. N., Van Lippevelde, W., & Overby, N. C. (2019). The Food4toddlers study - study protocol for a web-based intervention to promote healthy diets for toddlers: a randomized controlled trial. *BMC Public Health, 19*(1), 563. doi:10.1186/s12889-019-6915-x
76. Roset-Salla, M., Ramon-Cabot, J., Salabarnada-Torras, J., Pera, G., & Dalmau, A. (2016). Educational intervention to improve adherence to the Mediterranean diet among parents and their children aged 1-2 years. EniM clinical trial. *Public Health Nutr, 19*(6), 1131-1144. doi:10.1017/s1368980015002219
77. Salo, P., Viikari, J., Hamalainen, M., Lapinleimu, H., Routi, T., Ronnemaa, T., Seppanen, R., Jokinen, E., Valimaki, I., & Simell, O. (1999). Serum cholesterol ester fatty acids in 7- and 13-month-old children in a prospective randomized trial of a low-saturated fat, low-cholesterol diet: the STRIP baby project. Special Turku coronary Risk factor Intervention Project for children. *Acta Paediatr, 88*(5), 505-512. doi:10.1080/08035259950169503

Not-suitable intervention (n = 6)

78. Attanasio O, Baker-Henningham H, Bernal R, Meghir C, Pineda D, Rubio-Codina M. Early Stimulation and Nutrition: The Impacts of a Scalable Intervention. J Eur Econ Assoc. 2022;20(4):1395-432.
79. Ayer, J. G., Harmer, J. A., Xuan, W., Toelle, B., Webb, K., Almqvist, C., Marks, G. B., & Celermajer, D. S. (2009). Dietary supplementation with n-3 polyunsaturated fatty acids in early childhood: effects on blood pressure and arterial structure and function at age 8 y. *Am J Clin Nutr, 90*(2), 438-446. doi:10.3945/ajcn.2009.27811
80. Kemp, L., Harris, E., McMahon, C., Matthey, S., Vimpani, G., Anderson, T., Schmied, V., Aslam, H., & Zapart, S. (2011). Child and family outcomes of a long-term nurse home visitation programme: a randomised controlled trial. *Arch Dis Child, 96*(6), 533-

540. doi:10.1136/adc.2010.196279
81. Mihrshahi, S., Peat, J. K., Marks, G. B., Mellis, C. M., Tovey, E. R., Webb, K., Britton, W. J., & Leeder, S. R. (2003). Eighteen-month outcomes of house dust mite avoidance and dietary fatty acid modification in the Childhood Asthma Prevention Study (CAPS). *J Allergy Clin Immunol, 111*(1), 162-168. doi:10.1067/mai.2003.36
82. Muennig, P., Robertson, D., Johnson, G., Campbell, F., Pungello, E. P., & Neidell, M. (2011). The effect of an early education program on adult health: the Carolina Abecedarian Project randomized controlled trial. *American Journal of Public Health, 101*(3), 512-516. doi:10.2105/AJPH.2010.200063
83. Nct. (2009). Nutritional Education and the Prevention of Iron Depletion in Children 9 Months to 2 Years. *https://clinicaltrials.gov/show/NCT00907088*. Retrieved from https://www.cochranelibrary.com/central/doi/10.1002/central/CN-02024523/full

Non-eligible study design (n = 5)

84. Campbell, K. J., Abbott, G., Zheng, M., & McNaughton, S. A. (2017). Early Life Protein Intake: Food Sources, Correlates, and Tracking across the First 5 Years of Life. *Journal of the Academy of Nutrition & Dietetics, 117*(8), 1188-1197.e1181. doi:10.1016/j.jand.2017.03.016
85. Dotterud, C. K., Storro, O., Simpson, M. R., Johnsen, R., & Oien, T. (2013). The impact of pre- and postnatal exposures on allergy related diseases in childhood: a controlled multicentre intervention study in primary health care. *BMC Public Health, 13*, 123. doi:10.1186/1471-2458-13-123
86. Routi, T., Ronnemaa, T., Viikari, J. S., Leino, A., Valimaki, I. A., & Simell, O. G. (1997). Tracking of serum lipoprotein (a) concentration and its contribution to serum cholesterol values in children from 7 to 36 months of age in the STRIP Baby Study. Special Turku Coronary Risk Factor Intervention Project for Babies. *Ann Med, 29*(6), 541-547. doi:10.3109/07853899709007479
87. Spence, A. C., Campbell, K. J., Crawford, D. A., McNaughton, S. A., & Hesketh, K. D. (2014). Mediators of improved child diet quality following a health promotion intervention: the Melbourne InFANT Program. *Int J Behav Nutr Phys Act, 11*, 137. doi:10.1186/s12966-014-0137-5
88. Whaley, S. E., McGregor, S., Jiang, L., Gomez, J., Harrison, G., & Jenks, E. (2010). A WIC-based intervention to prevent early childhood overweight...Women, Infants, and Children. *Journal of Nutrition Education & Behavior, 42*(3 Supplement), S47-51. doi:10.1016/j.jneb.2010.02.010

Conferences - irrelevant topics (n = 3)

89. Anonymous. (2012). European Congress on Obesity, ECO2012. *Obesity Facts, 5*(SUPPL. 1). Retrieved from http://ovidsp.ovid.com/ovidweb.cgi?T=JS&PAGE=reference&D=emed13&NEWS=N&AN=70782569
90. Giesbrecht, G., Thomas-Argyriou, J., Salem, T., Johnstone, J., & Veugelers, P. (2019). Nutrition and mental health in children and youth. *Psychosomatic Medicine, 81*(4), A172. doi:http://dx.doi.org/10.1097/PSY.0000000000000699
91. Havas, S., Heimendinger, J., Damron, D., Nicklas, T. A., Cowan, A., Beresford, S. A. A., Sorensen, G., Buller, D., Bishop, D., Baranowski, T., & Reynolds, K. (1995). 5 A day for better health - Nine community research projects to increase fruit and vegetable consumption.. *Public Health Reports, 110*(1), 68-79. Retrieved from http://ovidsp.ovid.com/ovidweb.cgi?T=JS&PAGE=reference&D=emed5&NEWS=N&AN=25054267

Study ongoing (n = 2)

92. Blomkvist, E. A. M., Helland, S. H., Hillesund, E. R., & Overby, N. C. (2018). A cluster randomized web-based intervention trial to reduce food neophobia and promote healthy diets among one-year-old children in kindergarten: study protocol. *BMC Pediatr, 18*(1), 232. doi:10.1186/s12887-018-1206-8
93. De Franchis, R., Albano, F., Antignani, A., Bozza, L., Canale, P., Chiacchio, M., Cortese, P., D'Avino, A., De Giovanni, M., Iacovo, M. D., D'Onofrio, A., Federico, A., Gasparini, N., Iaccarino, F., Romano, G., Spadaro, R., Tedesco, M., Toscano, P., Vitiello, G., Auricchio, S., & Bruzzese, D. (2019). Taste development and eating habits of children following complementary feeding with adult food typical of the mediterranean diet. [Include]. *Journal of Pediatric Gastroenterology and Nutrition, 68*(Supplement 1), 1208. doi:http://dx.doi.org/10.1097/MPG.0000000000002403

Not-suitable Setting (n = 2)

94. Fard, N. M., Mehrabian, F., Sarraf-Zadegan, N., & Sajadi, F. (2004). Fat-modified diets during pregnancy and lactation and serum lipids after birth. *Indian J Pediatr, 71*(8), 683-687. doi:10.1007/bf02730653
95. Vitolo, M. R., Bortolini, G. A., Feldens, C. A., & Drachler Mde, L. (2005). [Impacts of the 10 Steps to Healthy Feeding in Infants: a randomized field trial]. *Cad Saude Publica, 21*(5), 1448-1457. doi:10.1590/s0102-311x2005000500018

Not-suitable control group (n = 1)

96. Melero V, Assaf-Balut C, de la Torre NG, Jimenez I, Bordiu E, Del Valle L, et al. Benefits of adhering to a mediterranean diet supplemented with extra virgin olive oil and pistachios in pregnancy on the health of offspring at 2 years of age. Results of the san carlos gestational diabetes mellitus prevention study. Journal of Clinical Medicine. 2020;9(5) (no pagination).

No full text (n=3):

1. Lee, P. (1988). The effects of a nutrition intervention programme on the nutritional status of pre-school children in disadvantaged areas of Dublin (PhD). Retrieved from https://www.cochranelibrary.com/central/doi/10.1002/central/CN-00735571/full
2. Isrctn. A health tool kit: primary prevention of obesity of preschoolers - the fit von klein auf randomised study in Lower Saxony, Germany. 2006.
3. Laitinen, K., Niinivirta, K., Nermes, M., & Isolauri, E. (2017). Relation of maternal dietary and probiotic intervention during pregnancy to the risk of atopic eczema and asthma in the offspring by the 4 years of age. *Allergy: European Journal of Allergy and Clinical Immunology, 72*(Supplement 103), 552. doi:http://dx.doi.org/10.1111/all.13252

Figure S1: Risk of Bias chart

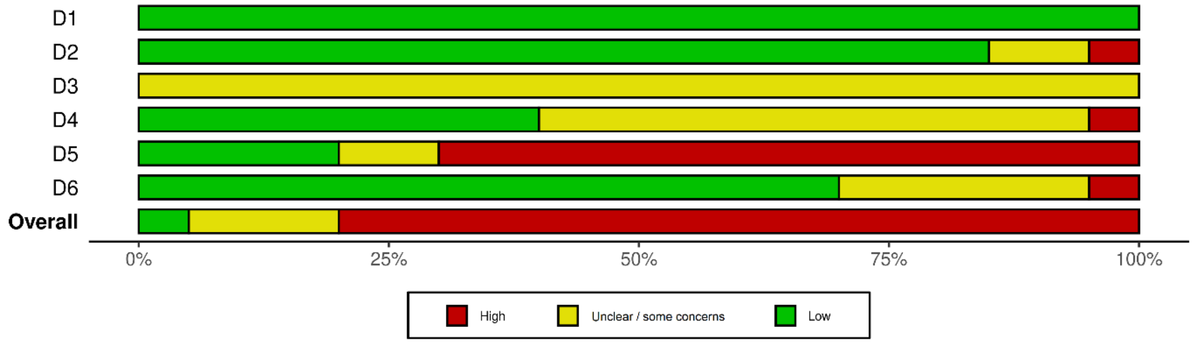

D1: Random sequence generation
D2: Allocation concealment
D3: Blinding of participants and personnel
D4: Blinding of outcome assessment
D5: Incomplete outcome data
D6: Selective reporting

Risk of Bias chart produced by the Cochrane RobVis tool (McGuinness and Higgins, 2020)
